# Supplementary material for: Are carbon emissions trading and green financial instruments synergistic? -Comprehensive quantitative research based on content analysis
Source: PLoS One. 2024 Mar 7;19(3):e0298601. doi: 10.1371/journal.pone.0298601 (PMC10919601; doi:10.1371/journal.pone.0298601)
Supplement: S1 Data — (ZIP) [file pone.0298601.s001.zip › Data/地级市Z_F工作报告.zip!/╝¬┴╓/░╫│╟╩╨/└┤╫╘╣1⁄2╓┌║┼í╛┬φ┐╦╩2╛▌═°í┐_2013.docx]

各位代表：

　　现在，我代表市政府，向大会作工作报告，请予审议，并请各位政协委员提出意见。

　　一、过去五年的工作

　　2008年以来，在省委、省政府和市委的正确领导下，在市人大、市政协的大力支持下，我们抢抓机遇，攻坚克难，拼搏奋斗，全面完成“十一五”规划，顺利实施“十二五”规划，圆满实现了市四届人大一次会议确定的各项目标，全面建设小康社会迈出了坚实的步伐、谱写了崭新的篇章。

　　——经济实力快速提升。预计2012年，全市地区生产总值完成650亿元，是2007年的2.8倍。固定资产投资完成450亿元，是2007年的2.4倍。全口径财政收入完成51亿元，是2007年的2.8倍；地方级财政收入完成32亿元，是2007年的3.8倍。三次产业结构由2007年的22∶41∶37调整为18∶49∶33。农业总产值完成217.5亿元，年均增长17.7%。规模以上工业增加值完成155亿元，年均增长25.1%。社会消费品零售总额完成220.6亿元，年均增长18.2%。初步形成了能源、农产品加工两大支柱产业，风电总装机规模达到310万千瓦，农产品加工企业发展到1195户，转化率达到50%以上。四个县（市）经济综合发展指数进入全省前30位。我市连续三年获得全省“发展杯”竞赛活动一等奖。

　　——改革开放不断深化。医药卫生体制改革、文化体制改革、事业单位分类改革稳步推进，国有粮食企业产权制度、集体林权制度改革全面完成，组建能源办、成立金融办。开发区扩权强区改革、“二次创业”成效显著，白城工业园区、通榆和镇赉开发区晋升为省级开发区，新建洮北开发区。“白纺”等17户企业完成重组。五年累计引进资金1770亿元，年均增长25%。己有5户世界500强企业、16户国内500强企业和9户央企入驻白城发展。建立中国与加拿大燕麦科技创新中心，已与76个国家和地区开展贸易往来、经济技术合作。

　　——人民生活明显改善。城镇累计新增就业23.3万人。城镇居民人均可支配收入由2007年11535元增加到19500元，年均增长11.1%；农民人均纯收入由2007年2851元增加到6200元，年均增长16.8%。新农保、城居保参保人数达28.8万人，“五险”覆盖面不断扩大。城市和农村人均住宅建筑面积分别达到27.5平方米、26.6平方米。互联网用户达到18万户，有线电视光缆入屯率达97%。243个村完成了扶贫整村推进，15.5万人实现脱贫，35万农村人口用上安全饮用水。

　　——城乡面貌显著变化。生态新城启动建设，老城区改造步伐加快，开发区基础设施建设力度加大。新建改造城市街路418公里，铺装巷道、小区573万平方米，新增绿地530万平方米。改造棚户区824万平方米，建设廉租房1.1万套，既有建筑节能改造774万平方米。县（市、区）污水处理厂全部建成使用。重点镇开发加快推进，399个新农村试点村镇建设成效明显。农村改造泥草房、危旧房28.2万户，砖瓦化率达到86%。珲乌高速公路白城段提前建成通车，新建改建等级公路4233公里，农村公路总里程达到8540公里，实现了“村村通”目标。

　　——生态建设富有成效。举市三年大造林完成造林120万亩，五年累计造林145万亩，森林覆盖率由10.9%提高到12%。种植生态草118.8万亩，改良草场500万亩。引嫩入白、大安灌区等水利工程发挥综合效益，引蓄过境水21亿立方米，恢复湿地1100平方公里。大力实施吉林西部土地开发整理项目，整理土地81万亩，荒漠化治理成效显著。我市被国家评为国土绿化突出贡献单位，被省评为十年绿化美化吉林大地先进市。大安嫩江湾被评为国家湿地公园，“嫩水韵白”获批国家水利风景区，镇赉被授予“中国白鹤之乡”称号，通榆“向海舞鹤”入选“吉林八景”前三甲。

　　——社会事业全面发展。争取国家、省科技发展计划项目203个，与国内73所大专院校、科技企业合作开发项目88个。城乡义务教育全面实现，建设农村标准化学校82所，新建改造校舍52.8万平方米，购置标准校车155台。公立医院改革深入推进，县级医疗机构、乡镇卫生院实行基本药物制度，社区卫生服务中心、村卫生室建设得到加强。在全省率先完成二轮续志工作。博物馆、群艺（文化）馆、图书馆免费开放。农村建成综合文化站73个、农家书屋918个、文化大院582个。广播电视实现了“村村通”。全民健身和竞技体育取得新成绩。低生育水平保持稳定，出生人口素质明显提高。全国双拥模范城创建实现“三连冠”。国家重大活动期间，实现进京非正常访、集体访“双零”目标。安全生产工作连续四年进入全省先进行列，实现无重大食品安全事故、无药害事件“双无”目标。群众安全感、满意度均达91%以上。民族宗教、外事侨务、妇女儿童、残疾人、老龄、气象、人防、地震、档案和红十字会等事业取得了新发展。

　　——自身建设切实加强。深入学习、践行科学发展观，不断提高履职能力。自觉接受人大、政协的监督，人大代表建议、政协委员提案办复率均达100%。圆满完成了市县两级政府机构改革任务。清理规范事业单位，市直撤销事业单位101个。减少行政审批项目203个。阳光政务“六项制度”深入实行。“12345”软环境建设、政风行风评议活动不断深化。“制度建设年”、“五讲”活动有效开展。绩效管理全面展开。“五五”普法较好完成，“六五”普法广泛开展。农村“三项制度”、政府性投资项目“制度+科技”监管机制、大安城市建设领域廉政风险“大联控”等做法在全省推广，反腐倡廉建设得到加强，政府公信力不断提高。

　　即将过去的2012年，我们面对经济下行压力和诸多困难，但仍然取得了可喜成绩。预计地区生产总值增长13.5%，固定资产投资增长25.7%，规模以上工业增加值增长15%，全口径财政收入增长20%、地方级财政收入增长30%，城镇居民人均可支配收入增长9.4%、农民人均纯收入增长12.5%。主要经济指标增速高于全省平均水平，地区生产总值、规模以上工业增加值、财政收入三项指标增速位居全省前列。

　　一年来，我们突出抓了八个方面工作。一是上项目扩投资。全市建设3000万元以上项目330个，其中亿元以上项目167个，10亿元以上项目20个，完成投资402亿元。金辉公铁立交桥竣工通车，长安机场启动建设，嫩丹高速全线征地、局部开工，白城内陆港一期主体工程完工，通榆500千伏输变电项目获国家核准。二是大招商增后劲。开展“招商上项目竞赛年”活动，启动五大区域驻点招商机制，依托43个商协会，联系554户企业，利用东博会等平台，广泛推介白城。全市引进项目835个，实际到位资金540亿元，增长25%。三是助企业稳增长。针对企业生产经营困难，全市抽调100名干部，深入100户企业，解决实际困难651项，争取专项补贴资金2000多万元，帮助贷款7亿元。争取高载能产业发展电价补贴政策，开展了富裕电力消纳试点。四是强基础兴农业。加快新开河治理、分洪入向等重点水利工程建设。发展膜下滴灌39.8万亩。粮食产量登上新台阶，有望突破100亿斤大关。举市三年大造林超额完成目标任务。实施新农村“富民强村固基”工程，启动100个示范村建设。五是活市场促消费。落实国家稳增长扩内需5个方面政策，加强“万村千乡市场工程”建设。举办白鹤节、垂钓节、辣椒节等特色活动。全市社会消费品零售总额增长16%。外贸进出口总额增长48%，增幅位居全省第一。六是抓城建强功能。完成生态新城规划，开工建设核心区基础设施和行政中心等重点项目。实施老城区改造提升十大工程，市区人行步道铺装88万平方米；投资5200万元，新建改造污水管网40公里，污水处理率由原来26.4%提高到60%以上。七是重投入惠民生。投资59.1亿元，完成年初确定的十个方面17件民生实事年度任务。积极争取国家和省专项资金7亿元，改造农村危房4.6万户，占今年全省改造总数的46%。八是破难题保稳定。化解信访积案150件，党的十八大和全国“两会”期间实现进京非正常访、集体访“双零”目标。工矿商贸、道路交通和火灾三类事故数量下降43.1%，强化食品药品监管，加强社会治安综合治理，刑事案件立案数下降11.6%。

　　各位代表！

　　五年来，我们准把市情谋发展，深挖资源增优势，抢抓机遇图振兴。这是科学发展的五年！

　　五年来，我们直面困难不回避，负重爬坡不懈怠，自我加压争上游。这是拼搏奋斗的五年！

　　五年来，市委统揽全局、协调各方，市人大、市政协鼎力支持，老领导老同志热情帮助，广大干部群众和衷共济。这是精诚团结的五年！

　　五年来，我们接力探索，找准了方向，喜获了成果，道路越走越宽广，生活越过越美好。这是信心倍增的五年！

　　五年的成绩来之不易。在这里，我代表市政府，向全市广大工人、农民、知识分子和各级干部、离退休老领导老同志，解放军驻白部队、武警官兵和公安干警，中省直各单位；向市人大、市政协、市法院、市检察院，各民主党派、工商联、人民团体和社会各界朋友，表示衷心的感谢！

　　总结过去的五年，我们也清醒地认识到，政府工作还存在许多不足，在发展道路上还面临着一些困难和问题。主要是：经济总量偏小、实力不强的状况还没有实质改变；资源开发程度浅，产业层次低，科技支撑弱的局面还没有明显扭转；制约发展的体制、机制和环境还有待于进一步优化；群众反映比较强烈的问题、影响社会和谐稳定的因素还不同程度存在；一些部门和干部作风不实、创新意识不强、办事效率不高的问题还没有根本解决；提升政府管理服务能力、加强廉政建设的任务还比较重，等等。对于这些问题，我们必须高度重视，认真加以解决。

　　二、今后五年的目标任务

　　党的十八大提出了全面建成小康社会和全面深化改革开放的目标，对新的时代条件下推进中国特色社会主义作出了全面部署，为社会主义现代化和中华民族的伟大复兴展现了更加壮丽的前景。省十次党代会对吉林科学发展、加快振兴进行了战略安排。市五次党代会对建设吉林西部特色经济区科学谋划，开启了白城富民强市、跨越发展的新征程。

　　今后五年，政府工作的总体要求是：深入学习贯彻党的十八大和中央经济工作会议精神，坚持以邓小平理论、“三个代表”重要思想、科学发展观为指导，全面落实省十次党代会、市五次党代会和市委五届五次全会部署，坚持“三化”统筹、“三动”并举，围绕建设吉林西部特色经济区，大力实施“三大战略”，深入推进“五大基地”建设、开发区“二次创业”，加快建设“六大功能区”，抓投资扩总量，调结构转方式，重生态优环境，惠民生促和谐，为实现白城富民强市历史性跨越、全面建成小康社会打下坚实基础。

　　今后五年的奋斗目标概括为：“四翻番”、“双倍增”、“六跃升”。

　　“四翻番”，就是地区生产总值、规模以上工业增加值、全口径财政收入、固定资产投资四项经济指标，在2012年基础上翻一番以上，分别突破1500亿元、500亿元、150亿元和1500亿元。

　　“双倍增”，就是城镇居民人均可支配收入、农民人均纯收入，在2012年基础上增加一倍，分别达到3.9万元和1.3万元。

　　“六跃升”，就是新型工业发展、清洁能源开发利用、特色精品农业生产、区域经济集聚能力、生态建设和环境保护、城乡建设和社会和谐发展跃上新台阶。

　　经过五年的奋斗，努力建设城乡经济繁荣、人民生活富裕、生态环境优良、社会和谐文明的吉林西部特色经济区。

　　实现上述奋斗目标，最重要的是抓住三大机遇，就是抓住国家加大粮食安全和食品安全投入机遇，加快提升我市粮食生产能力和农业产业化水平；抓住国家实施新一轮振兴东北老工业基地机遇，加快我市产业规划和转型升级；抓住国家启动新十年连片开发扶贫机遇，加快我市公共设施建设和扶贫攻坚进程。最根本的是挖掘三大资源，就是挖掘土地资源，拓展发展空间，增强农业效益；挖掘清洁能源资源，培育支柱产业，拉动工业快速增长；挖掘生态资源，打造绿色概念，发展生态产业。最关键的是破解三大瓶颈，就是破解“水”的瓶颈，改善农业设施和环境条件，振兴农业；破解“电”的瓶颈，解决电力消纳难题，振兴工业；破解“路”的瓶颈，改善区位劣势，振兴服务业。

　　今后五年的主要任务是：

　　（一）加快“三化”进程和转型升级，推动白城跨越发展。

　　实现快发展、大发展，必须坚持统筹推进、全面提升，始终保持强劲的发展势头。

　　着力做大工业。加快打造能源、农产品加工两大500亿元支柱产业，发展装备制造、冶金建材、汽车配套、纺织医药、生物化工五大100亿元优势产业。重点实施亿元以上工业项目100个、3000万元以上技改项目200个。通过战略重组和品牌打造，重点培育产值超50亿元企业3户、超10亿元企业30户、超亿元企业300户。加快开发区建设，打造10个产值超百亿元的特色园区。实施民营经济腾飞计划，扶持中小企业发展。到2017年，力争工业总产值实现1500亿元，年均增长27%。

　　着力做强农业。新增耕地170万亩，改造中低产田40万亩，保持粮食生产能力100亿斤以上。发展绿豆、葵花、蓖麻、辣椒及蔬菜、瓜果等高效经济作物，进一步优化种植结构。创建省级畜牧业园区，扶持1000个标准化规模养殖场，大力发展畜禽产业。实施品牌强农战略，加快发展有机、绿色、无公害和地理标志农产品，引进战略投资者开发白城野生鱼特色水产品，建设优质农产品基地。加快农田水利工程建设，发展棚膜、滴灌等现代设施农业，农业综合机械化水平提高到75%以上。推广示范性农民专业合作社500个，提升农业组织化程度。到2017年，力争农业总产值达到500亿元，年均增长18%。

　　着力提升服务业。开发大型商贸综合体，积极引进国内外商贸品牌企业。实施“万村千乡市场工程”，科学布局、改造提升城市市场，建设特色商业街区50个。发挥欧亚大陆桥和东北区域物流重要节点城市功能，培育建设特色农产品、农机、建材等集散交易中心和资源产品战略储备基地。提升房地产开发力度和品质，活跃房地产市场。壮大特色旅游业，强化对服务业的龙头带动作用。着眼老龄化社会的需求，发展社区家政服务业。积极培育信息中介、电子商务等新业态。到2017年，力争服务业增加值达到480亿元，年均增长14.8%。

　　着力加速城镇化。按照市场运作、滚动开发、产业支撑的原则，完成生态新城核心区建设，推进配套区基础设施建设，启动拓展区建设。围绕安全、整洁、有序的目标，坚持“六化”标准，持续推进老城区管网改造、街路整修、脏乱治理、社区建设和物业管理，提升老城区功能。壮大县域经济，加强基础设施建设，做优做特县城和中心镇。实施新农村“富民强村固基”工程，发挥100个示范村引领作用，带动乡村面貌实现较大转变。建设长安机场、嫩丹高速和长白快铁，构建城镇综合立体交通网络。探索户籍管理、土地流转、社会保障等制度改革，促进农业人口转移。到2017年，力争全市城镇化率达到55%。

　　着力推进扶贫攻坚。围绕全面建成小康社会的目标要求和国家大兴安岭南麓片区发展与扶贫规划，制定我市总体规划、年度计划和地区任务分解指标，全面打响扶贫攻坚战。立足资源优势，开发致富产业；立足环境改善，谋划设施项目；立足农民增收，出台优惠政策；立足解难济困，加大民生投入。大力实施农村居安工程，利用三年时间，全部改造农村危房。到2017年，实现35万农村贫困人口脱贫。

　　（二）增强内生动力和要素支撑，推动白城创新发展。

　　科学发展、后发赶超，必须坚持以改革激发新活力、以突破增强新优势。

　　培育新的增长点。一是突出发展现代高载能产业。通过财政补贴、减免税收、关联企业让利，对用电企业进行电价补贴，吸引现代高载能项目快速集聚，形成电力消耗、发电量增加、拉动装备制造业配套的产业发展模式。到2017年，年消耗电量达到100亿度，带动GDP增加500亿元，使高载能产业成为能源产业发展的引擎、工业振兴的突破口。二是突出推进农业产业化。依托农业资源优势，嫁接龙头企业。通过提升农业组织化程度，壮大基地规模，改善种养结构，培育特色品牌，提高农业精致化水平，延伸农业产业链条。到2017年，农产品加工转化率达到70%以上，使农业产业化成为农业现代化的重要途径、工业化的强大支撑和城镇化的重要动力。三是突出壮大特色旅游业。借助白城特有的生态、军事装备、地域文化优势，开发打造“湿地生态游”、“军事体验游”、“民俗风情游”三大品牌，建成全国特色旅游目的地。到2017年，全市接待旅游人数达到400万人次，旅游收入达到60亿元，使旅游业成为现代服务业的龙头、扩大对外开放的窗口。四是突出开发生态新城。通过高水准规划，提升城市品位；拉开城市框架，加快房地产开发；提升城市功能，发展现代服务业；营造生态环境，打造宜居新区。到2017年，完成核心区建设，使生态新城成为城镇化的加速器、城市经济的增长极。

　　深化改革开放。按照统一部署，抓好事业单位分类改革，推进收入分配制度改革，深化行政管理、财税、农村、医药卫生等重点领域和关键环节改革。积极融入东北“三省一区”区域经济发展格局，进一步拓展与东北亚各国及港澳台地区经贸往来，大力加强特色产品物流和出口基地建设。坚持大招商招大商，加强项目谋划，加大招商力度，完善招商机制，创新招商方法，把我市资源禀赋、政策优势与发达地区的资本、技术、品牌等优势进行最大程度地对接融合，提高对外开放合作的层次和水平。

　　加强科技支撑。围绕能源、农产品加工、汽车配套等重点产业，建立公共研发服务平台和产业技术战略联盟，服务技术创新。围绕资源特色和市场需求，借助外部科研力量，开展产品研发，走依靠科技创业之路。推进企业技术改造，加强企业信息化建设。加大科技兴农力度，充分发挥农科院、林科院、农机院、畜牧院优势，开展农业科技服务，强化实用技术推广。突出人才培养，加快经营管理人才、专业技术人才和农村致富能手三支队伍建设，进一步优化他们施展才华的环境和条件。

　　强化金融服务。吸引域外商业银行、保险业机构增设分支机构，组建农村商业银行，实现村镇银行县（市）全覆盖，做强中小企业担保公司，小贷公司力争发展到20家。加强上市培育，力争实现6户企业上市目标。开展涉农金融产品试点，探索金融服务“三农”新模式。完善政银企对接平台，深化“第一笔贷款”活动，扶持小微企业，促进实体经济发展。开展信用城市、信用企业创建，营造良好的金融环境。

　　用好政策资源。积极争取并充分用好国家集中连片扶贫开发区、国家振兴东北老工业基地重点规划城市、国家风电本地消纳示范区、吉林西部特色经济区4顶“金帽子”。围绕国家和全省的这些规划，找准切入点，融入区域发展大格局；用活差异化政策，在项目审批、产业升级、设施建设上争取更大的倾斜；抓住重点支持契机，破解发展瓶颈，解决历史遗留问题；借助政策新优势，打造“洼地”效应，吸引战略投资者；强化上争力度，在转移支付、政府投资、扶贫攻坚和民生投入等方面争取更多支持。

　　（三）强化生态建设和环境保护，推动白城绿色发展。

　　改善生态环境，是关系可持续发展的长远大计。必须把生态建设放在更加突出地位，努力建设美丽白城。

　　大兴植树造绿。以“一屏、二轴、三带、四景区、五群落”为总体布局，围绕第二个十年绿化美化吉林大地活动，实施三北百万亩人工林基地建设。以城乡绿化美化、公路绿色通道、城市出入口绿化、治沙治山治碱为重点，探索造林绿化新模式、社会融资新机制，推进苗木良种化、造林专业化、管理法制化、服务社会化，加快构建吉林西部绿色生态屏障。到2017年，全市完成造林150万亩，森林覆盖率达到14%。

　　加强湿地保护。推进向海自然保护区生态移民、长效供水、通道建设，理顺管理体制，从根本上破解向海湿地永续发展问题。加大莫莫格自然保护区保护与恢复力度，完善湿地补水配套工程和环境监控系统。推进嫩江湾国家湿地公园功能分区建设，培育全国知名的城市湿地品牌。提升“湿地风光、鹤乡白城”形象。

　　抓好草原建设。以国家实施草原补奖政策为契机，大力推进草原生态治理。采取草原围栏、草场改良、草原灌溉等措施，推行禁牧、休牧、轮牧制度，全面加强草原保护与建设，构建“草原增绿、牧业增效、牧民增收”发展格局。到2017年，力争草场改良100万亩，人工种草50万亩。

　　实施河湖连通。围绕建设引、蓄、灌、排相结合的现代化水利系统，利用引嫩入白、大安灌区、分洪入向等重大水利工程，加强配套工程建设，把江河洪水期富余水量引入境内141个可利用的天然湖泡，形成河湖互济、草茂粮丰、渔兴牧旺、水碧天蓝、人水和谐的水环境。

　　推进土地整理。科学编制土地整理规划，适时启动吉林西部土地整理二期工程。加大矿区、采砂、取土、油田废弃地复垦力度，提高植被覆盖率。采取田、水、路、林、村综合整治措施，推进高标准基本农田建设，改善生态环境。

　　强化环境保护。推进节能、节地、节水和资源综合利用，发展循环经济。重点防治火电、造纸、水泥、化工、农产品加工等行业污染，实现达标排放。改造污水处理厂，建设垃圾无害化处理场，加强城镇环境保护。围绕“清洁水源、清洁家园、清洁田园”的目标，开展生态村、生态镇和生态县创建活动。

　　（四）加强民生保障和社会建设，推动白城和谐发展。

　　人民安居乐业、社会和谐稳定，是我们一切工作的落脚点。必须坚持以人为本，大力发展民生事业，推动和谐社会建设。

　　扶持创业扩大就业。完善创业带动就业政策，抓好创业型城市试点，培育创业促就业基地20个，实施创业项目2000个。完善就业援助长效机制，重点解决高校毕业生、农村转移劳动力、城镇困难人员等群体就业问题。五年新增城镇就业23万人以上，保持城镇“零就业家庭”动态为零，农村劳动力转移就业175万人次。构建和谐劳动关系，维护劳动者权益。

　　加大社会保障力度。完善新农合制度，推进城镇基本养老、基本医疗、生育、失业和工伤五项社会保险全覆盖，新农保和城居保制度基本覆盖。发展社会福利和慈善事业，抓好困难群众救助救济工作，稳步提高保障标准和救助水平。实施城镇保障性安居、“暖房子”等工程，努力让城乡百姓住有所居、居所温暖。

　　促进文化繁荣发展。围绕文化育民，大力弘扬社会主义核心价值观，加强社会公德、职业道德、家庭美德和个人品德教育。围绕文化惠民，加快生态新城图书馆、博物馆、群众艺术馆、影剧院、奥林匹克公园建设，力争乡镇综合文化站、社区文化活动中心全覆盖。推进“三网融合”，丰富群众现代生活。围绕文化富民，规划建设仿古文化街区、国防文化基地、特色文化园区。加强城四家子城址等文化遗址保护。发展文化创意产业，着力培育白城文化名人。

　　协调发展社会事业。坚持教育优先发展，鼓励多种形式办学，改善办学条件，加强教师队伍建设，促进教育均衡发展。深化医药卫生体制改革，加强重点医院项目建设，健全农村三级卫生服务网络、城市社区卫生服务体系，提高医疗保障水平。广泛开展爱国卫生运动、全民健身活动。贯彻落实男女平等基本国策，稳定低生育水平。加强国防动员和后备力量建设，深入开展“双拥”共建活动。保障妇女儿童权益，加强未成年人保护。认真抓好第三次经济普查。发展老龄和残疾人事业，做好民族宗教、外事侨务、气象、人防、地震、档案、地方志和红十字会等工作，推动社会事业全面进步。

　　加强社会管理创新。实施“六五”普法规划，推进民主法制建设，提高依法治市水平。建立健全重大决策、重大项目社会稳定风险评估机制。实施“五有一创”工程，加强基层社会管理。完善信访制度，拓宽社情民意表达渠道。强化生产安全、消防安全、食品安全、工程安全管理，提高突发事件的预警预防和处置能力。深化平安白城创建工作，确保城乡居民安居乐业。

　　三、2013年工作安排

　　2013年是全面贯彻落实党的十八大战略部署的第一年，也是新一届政府的开局之年。站在新的起点上，面对世界经济进入深度转型调整期和我国经济发展重要战略机遇期，我们要因势利导，顺势而为，把握稳中求进、又好又快的工作总基调，扎扎实实开好局。

　　主要预期目标是：全市地区生产总值完成770亿元，增长13.9%。规模以上工业增加值完成185亿元，增长15%。全口径财政收入完成61亿元，增长20%；地方级财政收入完成38亿元，增长20%。固定资产投资完成590亿元，增长31.1%。社会消费品零售总额完成250亿元，增长13.3%。城镇居民人均可支配收入22500元，增长15.4%；农民人均纯收入7200元，增长16.1%。

　　围绕发展，着力抓好七项任务落实。一是抓好项目建设和招商引资。实施3000万元以上项目300个以上，其中新开工项目200个以上，实现竣工投产项目100个以上。深度谋划10亿元以上大项目100个，全年引进到位资金675亿元，增长25%以上。二是努力保持工业平稳增长。扶持20户骨干企业增产增效。培育30户成长型企业发展壮大。支持50户重点企业技改扩能。大力发展民营经济。三是推进富裕电力消纳。加快金升镍业、富达棒材扩能改造，抓好电解铝、太阳能电池组件、云计算、煤化工等高载能项目引进。四是加快开发区发展。进一步理顺管理体制，扩权强区。主要经济指标增幅必保达到30%，力争达到40%。五是提升农业产业化水平。制定农业产业化发展规划，推进农业产业化项目100个。粮食作物播种面积稳定在1200万亩。新建、改扩建标准化规模养殖场200个。建立完善100个农民专业合作社。六是加快发展现代物流和旅游业。启动建材家居、汽贸城等现代物流园区建设。抓好国家级特色农产品外贸转型升级示范基地项目申报。加快推进军事主题公园等25个旅游项目建设，实现旅游业收入30.6亿元，增长25%。七是推进农村扶贫开发。重点谋划实施一批带动力较强的产业扶贫开发项目、改善基本生产生活条件的基础设施建设项目，实行“市统筹、县落实”，建立项目“谋划、对接、调度”工作机制。实现7万农村贫困人口脱贫。

　　围绕建设，着力抓好四项任务落实。一是加强生态新城建设。推进9.6平方公里核心区建设，投资20亿元，完成配套水系建设，实施29条道路及管网、绿化景观和立交桥工程，推进行政中心、市民服务中心、科文中心建设。二是加强老城区改造管理。实施老城区绿化、管网改造、道路新建、街路亮化、巷道铺装等27个项目建设，着力打造1条标准化街路、1个市民广场、2个标准化小区。加强城市管理，开展“十乱治理”专项行动，加强社区建设和物业管理。三是加强基础设施建设。推进河湖连通工程，启动洮儿河、霍林河治理工程，加快分洪入向、新开河等中小河流治理工程建设。新打、改造抗旱水源井3000眼，完成膜下滴灌面积90万亩。开展新一轮大造林活动，力争完成造林40万亩。加快推进长安机场、嫩丹高速、嫩江公路大桥、长白快铁等项目建设。完成乡村公路建设200公里。四是加强县城和新农村建设。强化设施配套建设，拓展城区发展空间，推进县域突破。抓好新农村“富民强村固基”工程，全面启动示范村规划，推进重点项目建设，使城乡面貌实现新变化。

　　围绕民生，集中办好十个方面实事。一是全力做好就业工作。新增城镇就业4.6万人以上，促进1万名城镇下岗失业人员实现再就业。农村劳动力转移就业35万人次。二是提高社会保障水平。城镇居民医保实现全覆盖。扩大新农保、城居保覆盖范围。提高城乡低保、城镇居民医保补助标准。推进新农合按病种支付方式改革。三是实施城市保障性安居工程。改造城市棚户区211万平方米，建设廉租房15万平方米。四是加快“暖房子”改造。既有建筑节能改造185万平方米，管网改造152公里，撤并小锅炉40座。五是推进农村居安工程。改造农村危房3.4万户。六是保障农村饮水安全。解决5万农村人口饮水安全问题。七是改善城乡办学条件。维修改造校舍13.3万平方米。新建和改扩建公办幼儿园6所。新建农村标准化学校6所。八是提高医疗卫生服务能力。白城中心医院投入使用，办好县级医院，免费为2.7万名农村贫困妇女做“两癌”筛查。九是完善城乡文体设施建设。全民健身中心竣工投入使用。建设农村文化大院150个。送戏下乡400场。送电影到农村1万场。十是完善社会服务基础设施。新建92个农村社区服务中心。

　　围绕稳定，着力抓好五项任务落实。一是强化信访案件化解。深化领导干部接访下访，当年新发生信访案件结案率达到90%以上。二是强化安全生产隐患治理。突出危险化学品、烟花爆竹、道路交通、消防安全等8大重点行业领域，加大安全生产隐患治理力度，加强重点工程建设管理。三是强化社会治安防控体系建设。深入开展平安创建，落实“打防管控”综合措施。大型超市、商场、医院、学校等实现视频监控全覆盖。四是强化食品药品安全监管。严格准入、严格执法、严厉问责，保障人民群众饮食用药安全。五是强化公共突发事件预防处置。完善各项应急预案，强化信息报告预警，妥善处理各类突发事件，确保社会大局稳定。

　　四、加强政府自身建设

　　新的形势和任务，对政府工作提出了新的更高要求。我们要以建设服务型政府为目标，以提高政府执行能力为核心，全面加强政府自身建设，切实肩负起“富民强市”的历史重任。

　　（一）增强全局意识。自觉坚持党委的统一领导，紧紧围绕党委的战略部署，谋划工作、推进工作、落实工作。自觉接受和支持人大、政协的监督，坚持重大事项报告、通报制度，认真办理人大代表建议和政协委员提案。自觉执行重大决策集体研究、专家咨询、社会公示制度，广泛听取社会各界的意见和建议。

　　（二）强化创新观念。要开阔视野，开展到发达地区调研、考察、交流活动，在借鉴吸收中创新思维。要加强学习，系统研判宏观经济形势、产业发展趋势，在抢抓机遇中谋求发展。要营造氛围，鼓励创新、容忍失败，促进想干事、会干事、能干事的新人、能人脱颖而出。

　　（三）提高执行能力。严格按照法定权限和程序想问题、作决策、办事情，做到依法行政、规范有序。严格实行目标责任管理，细化量化任务，层层分解目标，层层抓好落实。要勤政高效，严谨细致，加强行政监察，强化绩效考核，严格行政问责，坚决治庸、治懒、治散，推动工作提速和效能提升。要攻坚克难，敢于碰硬较真，干事成事。

　　（四）坚持务实作风。严格执行中央关于改进工作作风、密切联系群众八项规定，自觉维护好、发展好、落实好广大群众的根本利益。深入开展“五讲”活动，继续实施阳光政务“六项制度”。加强“12345”软环境建设，开辟绿色审批通道，简化审批流程。开展民主评议政行风活动，打造优质高效服务环境。严格执行各项财经制度和纪律，严控行政开支，杜绝奢侈浪费。

　　（五）塑造廉明形象。推进政务公开从行政领域、较大公用企事业领域向全方位拓展，保障群众的知情权、参与权、表达权和监督权。全面落实“一岗双责”，深入推行政府性投资项目“制度+科技”监管、城市建设领域廉政风险“大联控”等有效机制，构建惩防体系，做到用制度约权、按制度办事、靠制度管人，从源头上预防腐败，坚持从严治政、从严治腐，以清正廉洁的形象取信于民。

　　各位代表！

　　实干成就宏伟事业，奋斗创造美好未来。让我们在党的十八大精神指引下，在省委、省政府和市委的正确领导下，在市人大、市政协的大力支持下，紧紧团结依靠全市各族人民和广大干部，解放思想，开拓创新，攻坚克难，奋发赶超，为实现白城富民强市历史跨越、全面建成小康社会而努力奋斗！
